# The Kinetics of Polymer Brush Growth in the Frame of the Reaction Diffusion Front Formalism

**DOI:** 10.3390/polym16212963

**Published:** 2024-10-23

**Authors:** Piotr Polanowski, Andrzej Sikorski

**Affiliations:** 1 Faculty of Chemistry, University of Warsaw, Pasteura 1, 02-093 Warsaw, Poland; 2 Department of Molecular Physics, Łódź University of Technology, Żeromskiego 116, 90-924 Lodz, Poland

**Keywords:** dynamic lattice liquid model, Monte Carlo method, polymer brushes, polymerization, reaction–diffusion front

## Abstract

We studied the properties of a reaction front that forms in irreversible reaction–diffusion systems with concentration-dependent diffusivities during the synthesis of polymer brushes. A coarse-grained model of the polymerization process during the formation of polymer brushes was designed and investigated for this purpose. In this model, a certain amount of initiator was placed on an impenetrable surface, and the “grafted from” procedure of polymerization was carried out. The system consisted of monomer molecules and growing chains. The obtained brush consisted of linear chains embedded in nodes of a face-centered cubic lattice with excluded volume interactions only. The simulations were carried out for high rafting densities of 0.1, 0.3, and 0.6 and for reaction probabilities of 0.02, 0.002, and 0.0002. Simulations were performed by means of the Monte Carlo method while employing the Dynamic Lattice Liquid model. Some universal behavior was found, i.e., irrespective of reaction rate and grafting density, the width of the reaction front as well as the height of the front show for long times the same scaling with respect to time. During the formation of the polymer layer despite the observed difference in dispersion of chain lengths for different grafting densities and reaction rates at a given layer height, the quality of the polymer layer does not seem to depend on these parameters.

## 1. Introduction

Polymer brushes are formed of polymer chains terminally attached to a surface. They have been a subject of numerous studies because of their practical importance for catalysis, drug delivery, optoelectronics, nanolithography, lubrication, and others [1,2,3,4]. Polymer brushes can be synthetized using two techniques: by tethering the chains that were already polymerized (“grafting to”) and by growing chains from initiators anchored on the surface (“grafting from”) [5,6,7,8,9,10]. The properties of polymer brushes were also determined using theoretical considerations [11,12,13,14,15,16,17,18,19,20] and computer simulations [11,21,22,23,24,25,26,27,28,29,30,31,32,33,34,35,36,37,38,39,40]. Recent simulation studies based on the Dynamic Lattice Liquid (DLL) model concerned the polymerization process and the dynamics of dense opposite brushes [41,42]. It was shown that there is a strong correlation between the dynamics of solvent and the local structure of the brush. The kinetics of the polymer brushes’ growth can also be regarded as a modified reaction diffusion front problem. The reaction diffusion front problem is usually formulated as follows [43,44,45,46,47]: Initially, an impenetrable barrier separates two kinds of species denoted as A and B. After the barrier is removed at time *t* = 0, reactants A and B start to form a reaction front. The reactant species mix before they react if their mobility is high enough in comparison with their reactivity. This leads to various kinetic regimes between the initial behavior and an asymptotic long-time behavior. In order to describe an irreversible reaction–diffusion process (A + B → 2C), where C is an inert product, the following mean-field-type equations for the concentration profiles *ρ_A_*(*x*,*t*) and *ρ_B_*(*x*,*t*) were used [48]:

(1a) $$\frac{\partial \rho_{A}}{\partial t}=D_{A}\frac{\partial^{2}\rho_{A}}{\partial x^{2}}-k\rho_{A}\rho_{B}\;$$

(1b) $$\frac{\partial \rho_{B}}{\partial t}=D_{B}\frac{\partial^{2}\rho_{B}}{\partial x^{2}}-k\rho_{A}\rho_{B}\;\;$$

where *D_A_* and *D_B_* are the diffusion coefficients of both species, while *k* is the microscopic reaction rate constant. Equation (1a,b) must satisfy the initial separation condition along the separation axis x:

(2a) $$\rho_{A}\left(x,0\right)=a_{0}\Theta \left(x\right)\;$$

(2b) $$\rho_{AB}\left(x,0\right)=b_{0}\left[1-\Theta \left(x\right)\right]\;$$

where a_0_ and *b_0_* are the initial concentrations of species A and B, and *θ*(x) is the Heaviside step function. The problem formulated in Equation (1a,b) was investigated in a real experiment [49,50,51], in theoretical considerations [48,52,53,54,55,56,57,58,59,60,61,62,63,64,65,66,67], and in computer simulations [68,69,70,71,72,73,74,75]. In the above considerations, it was assumed that diffusion is the only transport mechanism of molecular transport and that the diffusion coefficients were constant for all species regardless of the spatial location and concentrations of the reactants and products. This means that it was assumed that the correlation of movement between the A, B (reactants), and C (product) species was neglected. Such treatment can be considered as appropriate only in three cases: if the time range is short enough when compared to the inverse of the reaction rate (i), if the concentrations of species A and B are low enough, i.e., the correlation between the movements of the reactants and product is very weak (ii), and when the mobilities of all molecules are the same (iii). These conditions, however, are satisfied only in some exceptional systems, while in general, the mobilities of species are different and the correlation between their movements cannot be neglected in dense systems, especially for high concentrations of reactants. A correlation in motion of all species results in a variation in their diffusion coefficients within the reaction zone, which changes the kinetic behavior formulated in Equation (1a,b). In order to study more general cases, the problem has to be reformulated [76]. A correlation in the motions of species A, B, and C and the presence of an inert solvent, M, require the following extension of Equation (1a,b):

(3) $$\begin{array}{c} \frac{\partial \rho_{A}}{\partial t}=\frac{\partial }{\partial x}\left(D_{A}\left(x,t\right)\frac{\partial \rho_{A}\left(x,t\right)}{\partial x}\right)-k\rho_{A}\left(x,t\right)\rho_{B}\left(x,t\right)\\ \frac{\partial \rho_{B}}{\partial t}=\frac{\partial }{\partial x}\left(D_{B}\left(x,t\right)\frac{\partial \rho_{B}\left(x,t\right)}{\partial x}\right)-k\rho_{A}\left(x,t\right)\rho_{B}\left(x,t\right)\\ \frac{\partial \rho_{c}}{\partial t}=\frac{\partial }{\partial x}\left(D_{c}\left(x,t\right)\frac{\partial \rho_{c}\left(x,t\right)}{\partial x}\right)+k\rho_{A}\left(x,t\right)\rho_{B}\left(x,t\right) \end{array}$$

where *ρ_A_*(*x*,*t*), *ρ_B_*(*x*,*t*), and *ρ_C_*(*x*,*t*) are the local concentrations of reactants A and B, and products C, *D_A_*(*x*,*t*), *D_B_*(*x*,*t*), and *D_C_*(*x*,*t*) are the local diffusion coefficients. In the case of the polymerization process of a polymer brush composed of linear chains, two reactions occur:

(i) The chain initiation reaction

I + M → RI − End(4)

(ii) The chain propagation reaction

End + M → ….RM + End(5)

where I describes the initiator molecule, M is the monomer molecule from which the chain is built, RI is the reacted initiator, and End is the active end of the chain. In the reaction described in Equation (5), a new RM element is formed, which corresponds to the reacted monomer forming the polymer chain. It is obvious that in the case of the growth of polymer brushes grafted onto a surface, the reaction front will be formed by reacting chain ends and unreacted monomer molecules, while the product will be the elements of the chain. Thus, the propagating reaction front (active ends of chains) consumes reactants and leaves products behind (between the front and the surface).

In this paper, a coarse-grained model of brush synthesis was designed and studied. Due to complex architecture resulting from the large size and high density of polymer chains, these systems were studied by means of a lattice model. Fully flexible chains were immersed in a good solvent (monomer), and solvent molecules were explicitly included in the model. The process of polymerization was studied using the Monte Carlo method by means of the DLL model [77,78]. The “grafting from” approach was chosen, and thus, polymerization started from the initiator anchored to the surface, and all chains grew (the “grafting to” procedure does not enable the study of the polymerization process in dense brushes). Determining the influence of the reaction probabilities and the grafting density on the dynamic behavior of the reaction–diffusion front was the main goal of this study.

## 2. The Model and the Simulation Method

**Figure 1:**
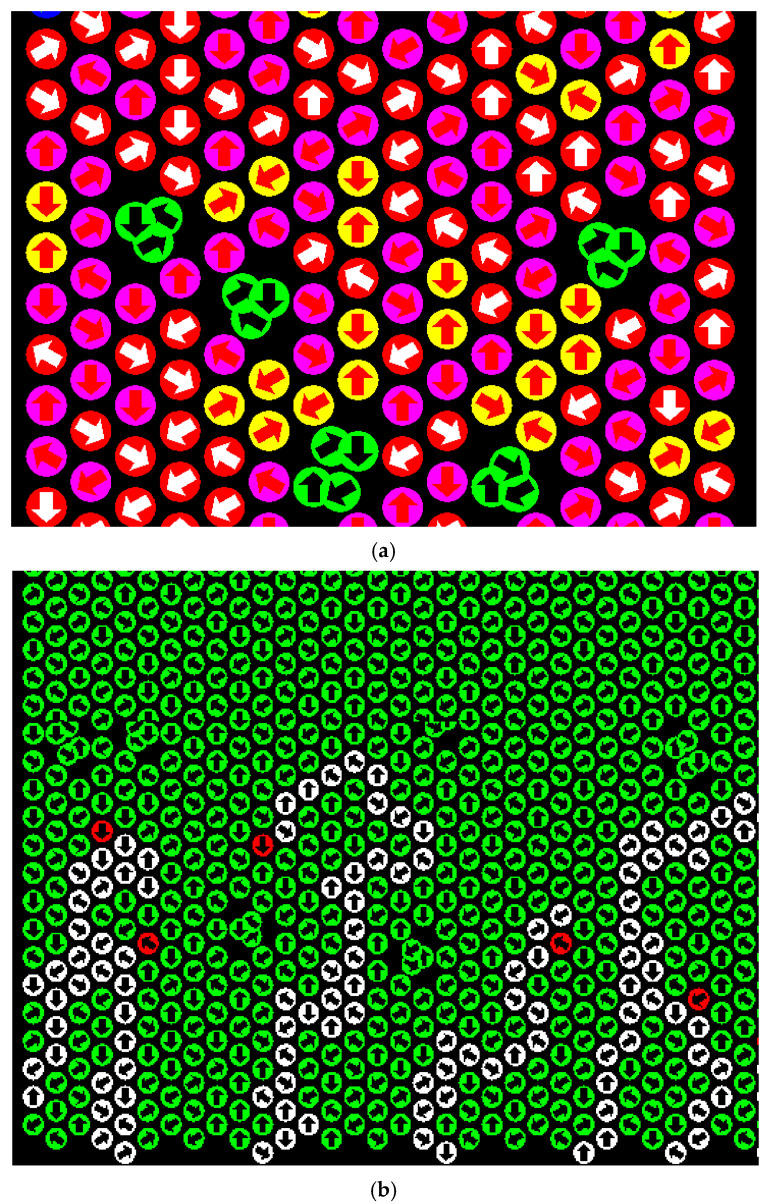
Panel (**a**): An example of a vector field representing attempts to move molecules towards neighboring lattice sites in the DLL model. The colored group of beads correspond to the following cases: a pair of beads trying to exchange places in the lattice (unsuccessful attempt, yellow), the attempt to move taking place from a point to which no other bead can achieve (unsuccessful attempt, violet), beads that do not properly form a cooperative loop (unsuccessful attempt, red), and each element exchanging its location with its neighbor (successful attempt, green). Panel (**b**): A cross-section in two dimensions of the process of creating brushes within the frame of the DLL method. In red are the active ends of the chains, in green is the unreacted monomer, and in white are the polymer chains. The arrows indicate attempts at achieving cooperative DLL movement.

Model chains were coarse-grained, and therefore, the macromolecule consisted of a linear sequence of beads. The positions of these beads and the beads representing the monomer molecules were restricted to the nodes of the face-centered cubic lattice (fcc). The simulation box consisted of 100 × 100 × 100 lattice sites. The layers x = 1 and 2 were immobilized and represented a hard flat surface. Positions of the initiator in these layers were selected at random, and the first polymer segment was thus usually located in the layer x = 3. At the beginning of each simulation run, the entire system was filled with monomer molecules. Periodic boundary conditions were used in all directions, so immobilized layers x = 1 and 2 acted as a reflecting surface in the x-direction. Monomers were attached to the initiator and then to the active ends of the chain, as described in Equations (4) and (5). The pseudo-bonds between the chain beads were fcc lattice vectors. Attachment of monomer occurred with specific probability of 0.02, 0.002, and 0.0002. The monomer is a good solvent for the polymer formed from it; therefore, it was assumed that the components of the system (monomer molecules and polymer beads) do not interact (athermal system). The dynamics of the system was realized using the DLL model. This model was described in detail elsewhere [79,80], and here, we include only a brief presentation in order to point out its main features. In one Monte Carlo step, a field of unit vectors representing motion attempts is randomly generated and assigned to all objects in the system. Only those attempts are successful, which coincide in such a way that the sum of displacements along a closed path (a cooperative loop) is equal to zero (the condition of continuity). In such a loop, each object is moved to a neighboring lattice site along this loop, while all objects that did not contribute to these loops stay in their previous positions (Figure 1a). Polymer beads can also participate in cooperative movement loops, provided that the bond in the chain is not broken during displacement. Polymer bonds are also impenetrable to liquid molecules. For longer times, this kind of dynamics leads to displacements of individual objects along random walk trajectories. The most important advantage of this method is that it naturally takes into account the heterogeneity of the system and introduces changes in the local mobility of the system components and steric hindrances resulting from the polymerization [81]. The DLL model suits conditions required for simulation of the polymer brush’s reaction front formation very well [76] due to both a good agreement with fundamental dynamical properties of liquids and a simple way of controlling mobilities of different constituents [82,83]. Moreover, this algorithm has already been successfully used several times in the study of double polymer brushes [41,42], polymer brushes grafted onto surfaces of different geometries [34,84,85], and copolymer branched brushes [86]. The scheme in Figure 1b shows the process of building chains using the DLL algorithm.

The polymerization process was monitored by the following commonly used quantities:

(i) The averaged degree of polymerization

(6) $$DP_{n}=\frac{\sum_{i}n_{i}p_{i}}{\sum_{i}p_{i}}\;$$

where *n_i_* corresponds to the given chain length and *p_i_* describes the fraction of chains with the length *n_i_*.

(ii) The weight-averaged degree of polymerization defined asn

(7) $$DP_{w}=\frac{\sum_{i}n_{i}^{2}p_{i}}{\sum_{i}n_{i}p_{i}}$$

(iii) The distribution of the chain length is characterized by the dispersity *D* that is defined as the ratio $\frac{DP_{w}}{DP_{n}}$.

(iv) The parameter that describes the structure of the entire brush is the mean brush thickness <*x*>, defined as [87]

(8) $$\langle x\rangle =\frac{\sum_{x=1}^{x_{max}}p\left(x\right)x}{\sum_{x=1}^{x_{max}}p\left(x\right)}$$

where *p*(*x*) is the polymer density along the direction normal to the grafting surface, and xmax is the maximum distance of the polymer bead form the surface. In order to describe the dynamic behavior of the reaction–diffusion front during brush polymerization, the following parameters are considered: the local production rate *R*(*x*,*t*), the width of the front w(t), and the height of the front *R*(*x_f_*,*t*). The local production rate that results in the growth of polymer chains in the reaction described in Equation (5) can be described as

(9) $$R\left(x,t\right)=k\cdot \rho_{End}\left(x,t\right)\cdot \rho_{M}\left(x,t\right)$$

where *ρ_End_*(*x*,*t*) corresponds to a local density of active chain ends and *ρ_M_*(*x*,*t*) is a local density of monomers, and *k* is the reaction rate coefficient. In this work, we use a reaction probability *p* instead of reaction rate based on our previous study [76]. The *R*(*x*,*t*) function describes the local kinetics, but it is a basis of the dynamic behavior of the entire system. The spatial integration over *R*(*x*,*t*) leads to the following:

(10) $$R\left(t\right)=\sum_{x}R\left(x,t\right)\;$$

*R*(*x*,*t*) is called the global production rate, which describes the reaction rate at the macroscopic level. The position of the center of the reaction front x_f_(*t*) is defined as a position where the *R*(*x*,*t*) function reaches its maximum. The width of the front *w*(*t*) is described by the following equation:

(11) $$w^{2}\left(t\right)=\frac{\sum_{x}{\left(x-x_{f}\left(t\right)\right)}^{2}R(x,t)}{\sum_{x}R(x,t)}$$

And the height of the front (local rate at *x_f_*− *R*(*x_f_*,*t*)) is the last important quantity describing the diffusion reaction front.

## 3. Results and Discussion

**Figure 2:**
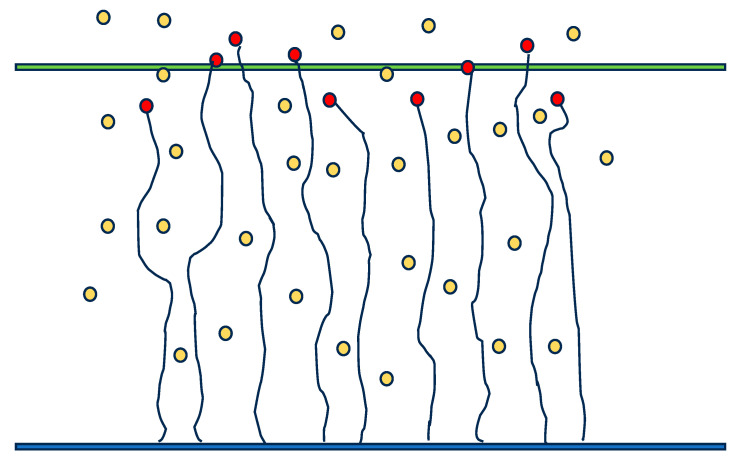
A scheme of the reaction front in a brush growing from the surface (blue). The red circles mark active chain ends, the yellow circles mark unreacted monomer molecules, and the green line shows approximately the middle of the reaction front.

The grafting density, GD, was defined as the ratio of the sites occupied by the initiator on the plane to the area of that plane (100 × 100). Simulations during which brush growth was observed were performed for GD = 0.1, 0.3, and 0.6. The polymerization reaction, i.e., the attachment of a monomer to a growing chain, can take place if the end of the chain and the monomer in question occupy adjacent positions on the lattice but with a certain assumed probability. Simulations were carried out for the following probabilities of polymerization: *p* = 0.02, 0.002, and 0.0002. A scheme of a reaction front in a growing brush is presented in Figure 2.

### 3.1. The Properties of the Obtained Brushes

**Figure 3:**
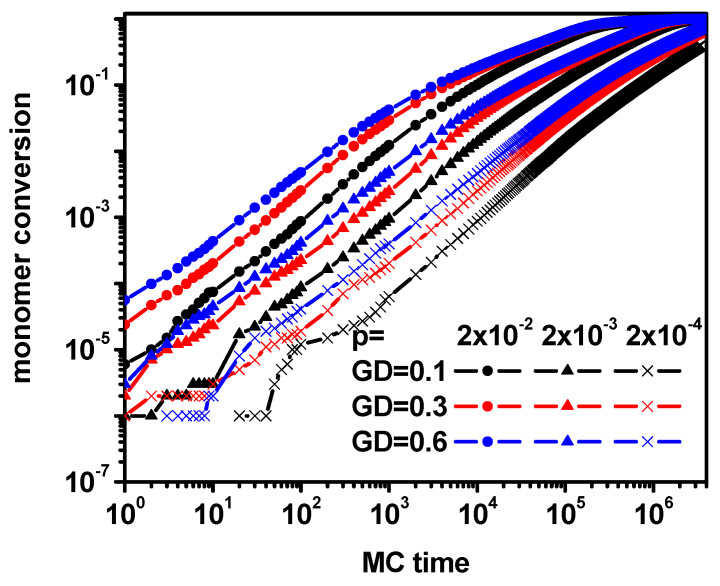
Monomer conversion for various grafting densities and the probability of chain propagation as a function of time. The values of GD and *p* are given in the inset.

First, the changes in the monomer conversion rate with time (the number of Monte Carlo steps) were investigated for several grafting densities and several probabilities of polymerization reaction occurrence. Figure 3 shows monomer conversion as a function of time for some grafting densities and reaction probabilities.

**Figure 4:**
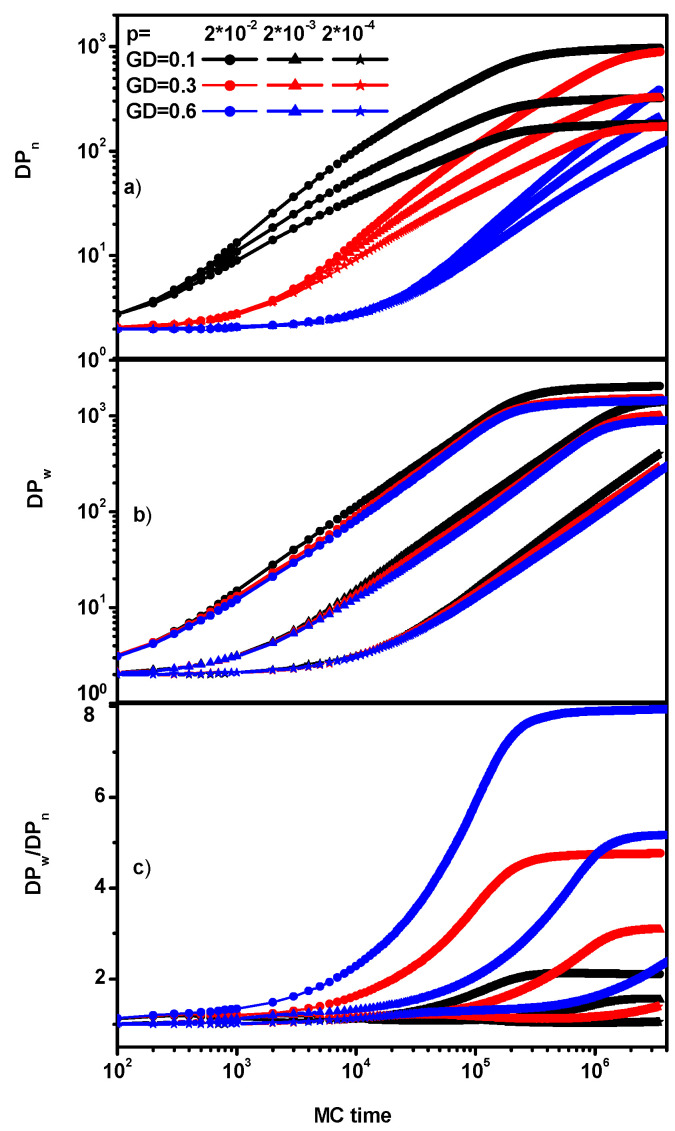
The averaged degree of polymerization *P_n_* (**a**), the weight-averaged degree of polymerization *P_w_* (**b**), and dispersity (**c**) as a function of time. The values of GD and *p* are given in the inset.

One can observe that all curves are similar in the early stages of synthesis. Later on, monomer conversion becomes slower, especially for high grafting densities and higher reaction probabilities. As expected, the rate of monomer conversion is the highest for the highest reaction probabilities and the highest grafting densities due to the number of active chain ends (the higher the grafting density, the more active chain ends present). Due to the observed differences in monomer conversion, two types of characteristics regarding chain mass and dispersion may be useful in studying the brush growth process: against time and against the degree of monomer conversion. Figure 4 shows the changes in both degrees of polymerization, *P_n_* and *P_w_*, as well as in *P_w_*/*P_n_*, which can be treated as a measure of chain dispersion as a function of time (dispersity).

In Figure 4, it can be observed, as expected, that the average mass of the chain *P_n_* grows the fastest in the case of the smallest grafting density, which is obvious, and it depends on the reaction rate (for high reaction rates, it increases). It is possible to distinguish three phases in the process of brush synthesis: the first one in which the growth is slow, the second one, which has fast growth, and the third one in which the polymer mass reaches a plateau, which is connected with geometrical limitations of the system. The brush does not grow because it has reached the maximum height (it should be noted here that the brush does not grow despite the fact that the whole monomer has not been exhausted because the ends of chains do not have access to it as it is located in lower layers and ends cannot react with it). It is worth noting that the dispersion is considerably lower with decreasing grafting density and reaction rate, indicating that the resulting polymer layer is more homogeneous. It is important to note that the situation we are considering takes place during brush growth, i.e., during a non-equilibrium process.

The changes in the parameters discussed above, i.e., *P_n_*, *P_w_*, and *P_w_*/*P_n_*, can also be studied depending on the degree of monomer over-reactivity.

**Figure 5:**
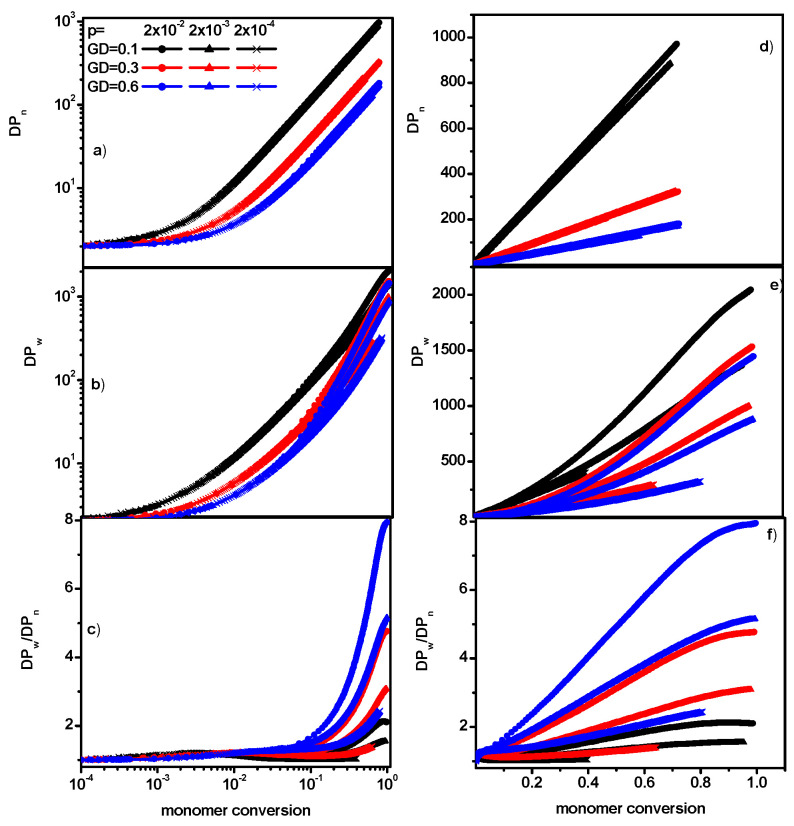
The average degree of polymerization *P_n_* (**a**,**d**), the weight-averaged degree of polymerization *P_w_* (**b**,**e**), and dispersity (**c**,**f**) as a function of monomer conversion. The values of GD and *p* are given in the inset. The semi-log plot is on the left, and the linear plot is on the right.

Figure 5 shows *P_n_*, *P_w_*, and dispersity as a function of monomer conversion. It can be seen that for a given grafting density, irrespective of the reaction rate, the average mass of the chains increases in the same way as the monomer reactivity increases. The maximum molecular weight (the chain length) after the polymerization process is completed decreases with the grafting density: the number of chains is higher, while the amount of the monomer available in the system remains the same. The dispersity is considerably higher for higher grafting densities and for a low reaction probability. For higher values of the reaction probability and low grafting densities, the differences in dispersity remains small during almost the entire polymerization process.

**Figure 6:**
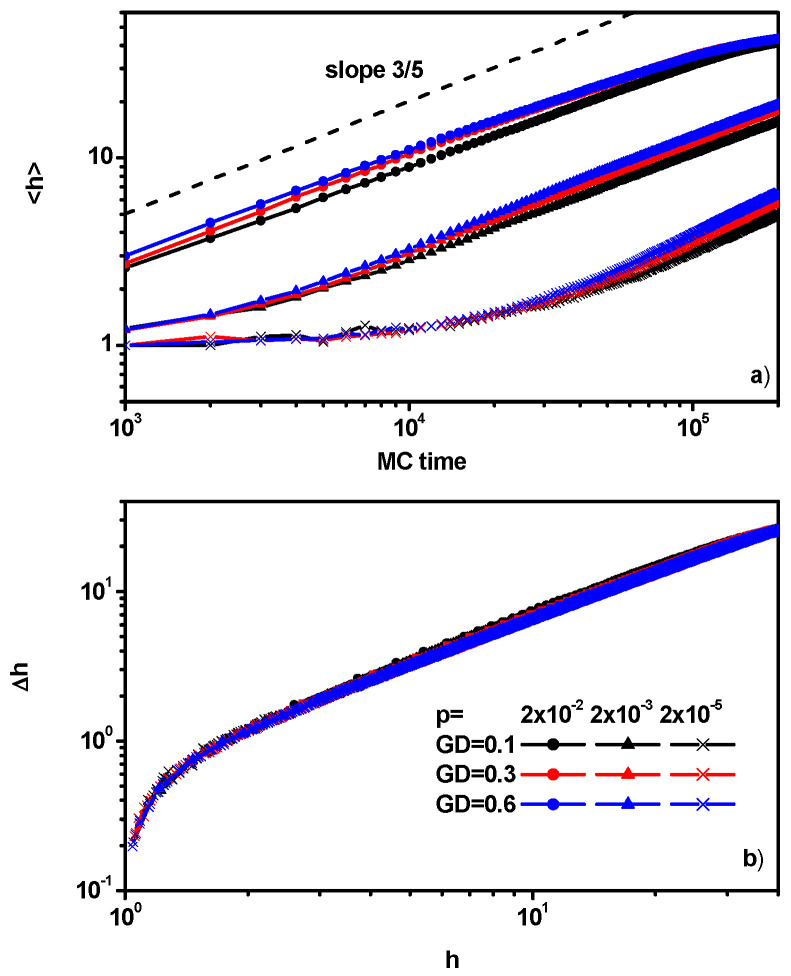
The mean height of the brush as a function of time (**a**) and the deviation from the mean brush height as a function of the brush height (**b**). The values of GD and *p* are given in the inset.

The above results can be confirmed by the behavior of the brush height <*h*> with time. Figure 6a shows the changes in the mean brush height as a function of time in the considered range of grafting density and the chain propagation probability. The growth rate of the brush height decreases with a decreasing reaction rate; however, changes in height over time show a certain universality, i.e., the mean value of the brush height approximately shows a similar variation in time <*h*>~*t*.

A good measure of polymer layer homogeneity is the deviation from the mean value of the brush height:

(12) $$∆h=\sqrt{\frac{\langle h^{2}\rangle -{\langle h\rangle }^{2}}{{\langle h\rangle }^{2}}}$$

Figure 6b presents the deviation Δ*h* as a function of the brush height. The average deviation Δ*h* can be taken as a measure of the quality of the polymer layers: a small value of Δ*h* means that the layer (the brush) is less rough. One can see that regardless of the grafting density and the reaction probability, the deviation Δ*h* is practically the same for a given brush height. Thus, we see that the dispersion value alone, presented above in Figure 4c, does not tell us the quality of the polymer layer.

### 3.2. Reaction–Diffusion Front

In the classical case in which the reaction front problem is considered [48], the densities of the reacting substrates on both sides of the front are the same, and the diffusion coefficients appearing in Equation (1a,b) are also the same. Galfi and Racz [48], using arguments based on a scaling theory, obtained some surprising results, the most unexpected of which was concerning the scaling of the width of the reaction front for long times, *w*(*t*)~*t^α^*, where *α* = 1/6 and the reaction rate at the center of the front *R*(*x_f_*, *t*) ranges with time as *t*^−2/3^. Additional studies based on the perturbation theory [72] have found time dependencies for reaction fronts over the entire time range, and the results are presented in the Table 1.

The case described above is a special case in which, due to the equality of substrate concentrations and mobility, the position of the front’s center does not change. In the general case where such conditions are not met, the center of the front of the system may shift [63].

**Figure 7:**
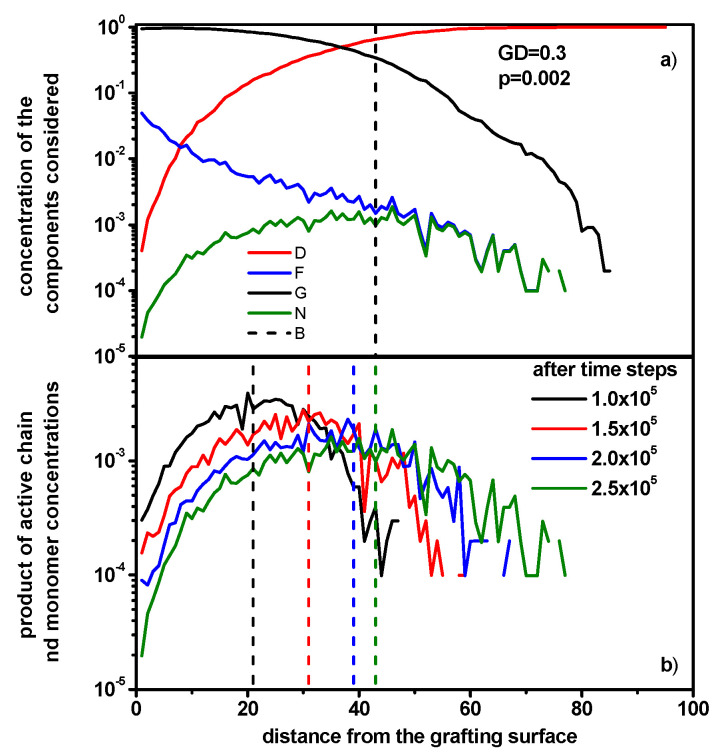
The concentration of the system components in the direction perpendicular to the grafting surface for DG = 0.3, and *p* = 0.002 after 2.5 × 10^5^ simulation steps. The black line denotes the concentration of polymer beads, the red line denotes the concentration of unreacted monomer, the blue line indicates the concentration of active chain ends, and the green line indicates the concentration of products of active chain ends and the concentration of unreacted monomer (**a**). The concentration of products of active chain ends and the concentration of unreacted monomer for DG = 0.3 are shown, and *p* = 0.002 after various simulation steps. The dashed lines indicate the positions of the reaction diffusion front center *x_f_* (**b**).

Figure 7a shows the concentrations of the components in the system in which the brush is synthetized after 2.5 × 10^5^ simulation steps. Due to the differences in the mobility and concentration of the monomer and active ends of the chain, a clear shift in the center of the reaction front in the direction opposite to the grafting surface can be seen, as illustrated by Figure 7b in which the positions of the reaction front center for the indicated times are shown by vertical dashed lines.

**Figure 8:**
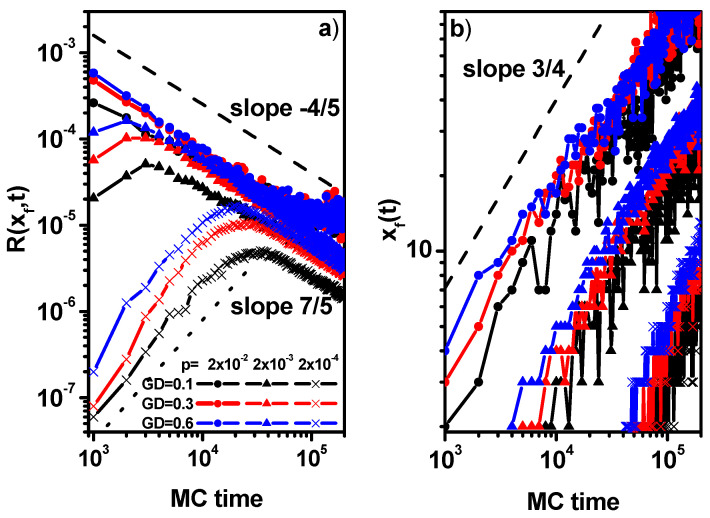
The reaction rate at the reaction front center as a function of time (**a**). The position of the reaction front center as a function of time (**b**). The values of GD and *p* are given in the inset.

Figure 8a shows the reaction rate at the center of the front. For long times, it decreases with time as *R*(*x_f_*,*t*)~*t*^−4/5^, which is much faster than in the classical case where the scaling exponent is −2/3 [72]. For small reaction probabilities of 0.0002, one can see the exact short-time regime in which the reaction rate increases very rapidly as *R*(i,*t*)~*t*^7/5^. Figure 8a also suggests that the change from the short-time regime to the long-time regime is shifting towards shorter times with increasing grafting density and reaction rate. The position of the center of the reaction front *x_f_* is presented in Figure 8b. It is clearly seen that the rate of the shifting front increases with the reaction rate and is weakly dependent on the grafting density (it seems to be higher for larger inoculations) but, irrespective of the grafting density, the scaling is approximately the same with time, i.e., *x_f_*~*t*^3/4^.

**Figure 9:**
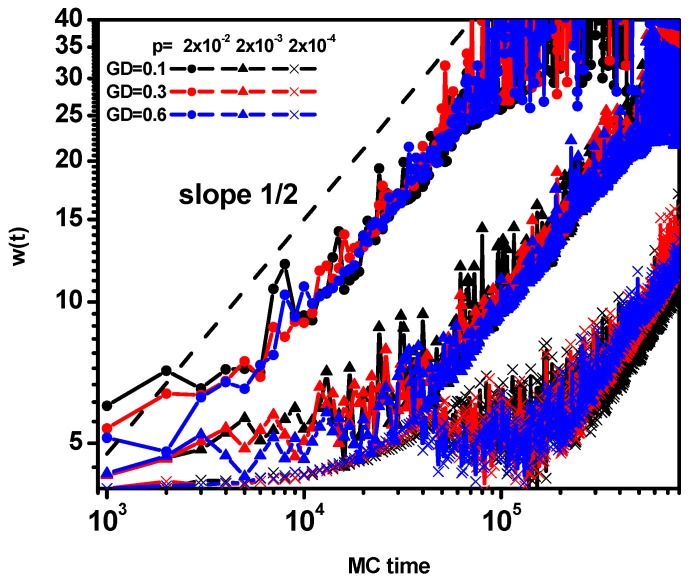
The reaction diffusion front width *w*(*t*) as a function of time. The values of GD and *p* are given in the inset.

Figure 9 shows the time dependence of the reaction front width *w*(*t*). The width of the reaction front during the synthesis process and the growth of the polymer brush increases with an increasing reaction rate and approaches approximately the same values regardless of the grafting density. As in the case of the reaction rate in the center of the front, in the case of the width of the front, we observe a certain universality in the form of a similar increase in time manifested by a scaling of *w*(*t*)~*t*^1/2^; this relationship is much stronger than in the classical case for small reaction rates as one can also observe a short-time regime in which the front grows very slowly.

**Figure 10:**
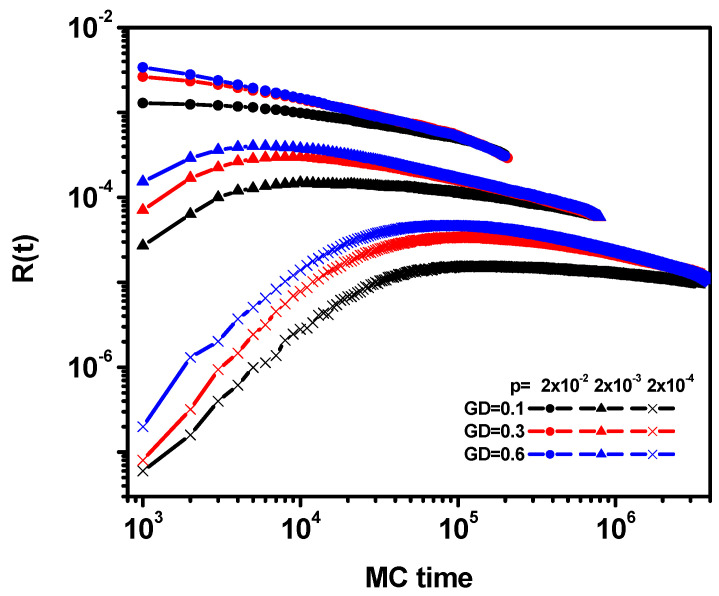
The reaction *R*(*t*) rate as a function of time. The values of GD and *p* are given in the inset.

The changes in the reaction rate *R*(*t*) over time are presented in Figure 10. The reaction rate depends on the two parameters considered here, that is, the reaction probability and the grafting density. Note, however, that in comparison, the dependence on the former is significantly greater, and with an increasing number of Monte Carlo steps, the dependence on grafting density disappears. The fact that R(t) increases as the reaction probability increases is understandable. However, it is also the highest in systems with the highest grafting density. This is because a large number of chains (and therefore active ends) attach more monomer molecules. In the case of this parameter, two regimes are also visible: for a short time, *R*(*t*) increases, while for a long time, it decreases (with the exception of a high probability of polymerization, *p* = 2 × 10^−2^, where there is no regime with an increase in *R*). It is difficult to determine the behavior in the long term because, as one can see, these curves asymptotically approach each other, but it is impossible to carry out any scaling, although it possible that all curves converge to the scaling close to the theoretical *t*^−1/2^ value [72].

## 4. Conclusions

The process of polymer brush formation was studied using Monte Carlo computer simulations. A suitable coarse-grained and lattice model of the brush was designed, and its properties were determined within a Dynamic Lattice Liquid framework. Brush polymerization was carried out in a system completely filled with monomer with the initiator placed randomly on an impenetrable surface. The effects of the polymerization reaction rate (the probability of the reaction) and chain grafting density on the structure and quality of the resulting polymer layer and on the properties of the reaction front were studied.

The parameters describing the brush were studied during the polymerization process. It turned out that, despite the differences in the dispersion of chain lengths, the brush increases in the same way, i.e., the mean deviation of the brush height does not depend on the grafting density and the reaction rate. Within the formalism of reaction fronts, we observed universality in the width of the front and height of the front: for long times, they exhibit the same exponents. In general, the polymer layers are more homogeneous at large grafting densities and small probabilities of polymerization reaction. So, we can show how the quality of the polymer layer, which is a polymer brush, can be controlled with selected parameters in order to obtain the quality and homogeneity of the surface coating. In addition to the practical issue, there is an important aspect related to the development of the theory of non-equilibrium processes. The process of reaction front formation can be considered as an example of the simplest non-equilibrium process. In the work presented here, a study of the formation and propagation of the reaction front in the case of polymer brushes was carried out, and new scaling was obtained, which seems to be a contribution to the theory of reaction fronts and indicates that, in this case, the behavior of the front deviates from that previously observed.

## Figures and Tables

**Table 1:** The scaling exponents of parameters describing the reaction–diffusion front (from Ref. [72]).

Quantity	Short-Time Behavior	Long-Time Behavior
R(t)	t^1/2^	t^−1/2^
W(t)	t^1/2^	t^1/6^
R(xf, t)	const	t^−2/3^

## Data Availability

The original contributions presented in the study are included in the article, further inquiries can be directed to the corresponding author.
